# 7-Azido-*N*,*N*-diethyl-4,5-*O*-isopropyl­idene-4-*C*-methyl-3,6-anhydro-7-de­oxy-d-*glycero*-d-*manno*-heptonamide

**DOI:** 10.1107/S1600536808044279

**Published:** 2009-01-08

**Authors:** Sarah F. Jenkinson, Chen Wang, Maria-Soledad Pino-González, George W. J. Fleet, David J. Watkin

**Affiliations:** a Department of Organic Chemistry, Chemistry Research Laboratory, Department of Chemistry, University of Oxford, Oxford OX1 3TA, England; b Departamento de Bioquímica, Biología Molecular y Química Orgánica, Facultad de Ciencias, Universidad de Málaga, 29071 Málaga, Spain; c Department of Chemical Crystallography, Chemistry Research Laboratory, Department of Chemistry, University of Oxford, Oxford OX1 3TA, England

## Abstract

The reaction of 5-azido-5-de­oxy-2,3-*O*-isopropyl­idene-2-*C*-methyl-d-ribose with *N*,*N*-diethyl-2-(dimethyl­sulfuranyl­idene)acetamide gave the title compound, C_15_H_26_N_4_O_5_, as the major product arising from initial formation of an epoxide which was subsequently opened by intra­molecular attack of the free 4-hydroxyl group. X-ray crystallography confirmed the relative stereochemistry of the title compound and the absolute configuration was determined by the use of d-ribose as the starting material. The crystal structure contains chains of mol­ecules running parallel to the *a* axis, being linked by weak bifurcated O—H⋯(N,N) hydrogen bonds.

## Related literature

For related literature see: Assiego *et al.* (2004 ▶); Pino-González *et al.* (2003 ▶, 2008 ▶); Valpuesta Fernández *et al.* (1990 ▶); Valpuesta *et al.* (1993 ▶); Görbitz (1999 ▶).
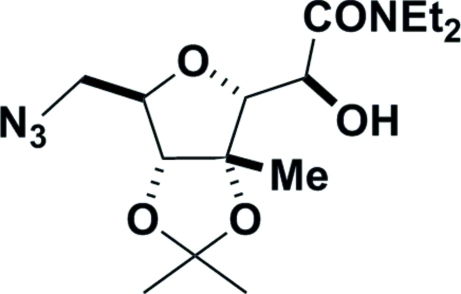


## Experimental

### 

#### Crystal data

C_15_H_26_N_4_O_5_
*M* *_r_* = 342.40
Orthorhombic, 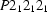

*a* = 8.64400 (10) Å
*b* = 13.4195 (2) Å
*c* = 15.9146 (3) Å
*V* = 1846.06 (5) Å^3^
*Z* = 4
Mo *K*α radiation
μ = 0.09 mm^−1^
*T* = 150 K
0.60 × 0.60 × 0.40 mm

#### Data collection

Area diffractometer
Absorption correction: multi-scan (*DENZO*/*SCALEPACK*; Otwinowski & Minor, 1997 ▶) *T*_min_ = 0.82, *T*_max_ = 0.96
23123 measured reflections
2354 independent reflections
2077 reflections with *I* > 2σ(*I*)
*R*_int_ = 0.077

#### Refinement

*R*[*F* ^2^ > 2σ(*F* ^2^)] = 0.049
*wR*(*F* ^2^) = 0.129
*S* = 1.02
1992 reflections
217 parameters
H-atom parameters constrained
Δρ_max_ = 0.24 e Å^−3^
Δρ_min_ = −0.20 e Å^−3^

### 

Data collection: *COLLECT* (Nonius, 2001 ▶); cell refinement: *DENZO*/*SCALEPACK* (Otwinowski & Minor, 1997 ▶); data reduction: *DENZO*/*SCALEPACK*; program(s) used to solve structure: *SIR92* (Altomare *et al.*, 1994 ▶); program(s) used to refine structure: *CRYSTALS* (Betteridge *et al.*, 2003 ▶); molecular graphics: *CAMERON* (Watkin *et al.*, 1996 ▶); software used to prepare material for publication: *CRYSTALS*.

## Supplementary Material

Crystal structure: contains datablocks global, I. DOI: 10.1107/S1600536808044279/lh2750sup1.cif

Structure factors: contains datablocks I. DOI: 10.1107/S1600536808044279/lh2750Isup2.hkl

Additional supplementary materials:  crystallographic information; 3D view; checkCIF report

## Figures and Tables

**Table 1:** Hydrogen-bond geometry (Å, °)

*D*—H⋯*A*	*D*—H	H⋯*A*	*D*⋯*A*	*D*—H⋯*A*
O17—H171⋯N10^i^	0.88	2.30	3.112 (4)	152
O17—H171⋯N11^i^	0.88	2.45	3.313 (4)	167

Symmetry code: (i) 
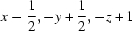
.

**Fig. 1.**
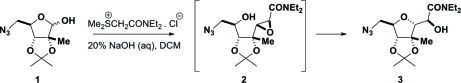
Synthetic Scheme

**Fig. 2.**
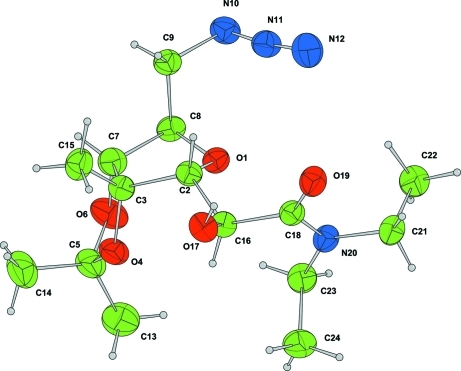
The title compound with displacement ellipsoids drawn at the 50% probability level. H atoms are shown as spheres of arbitrary radius.

**Fig. 3.**
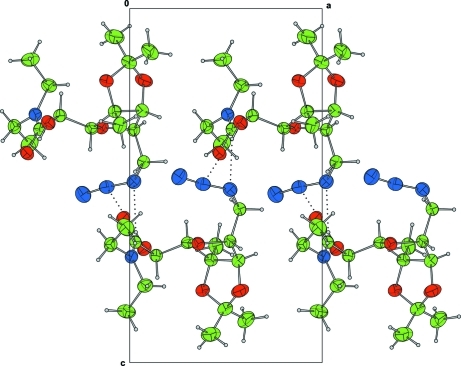
Packing diagram for the title compound projected along the b-axis. Hydrogen bonds are indicated by dotted lines.

## supplementary crystallographic information

### Comment

The use of sulfur ylids in the stereoselective formation of epoxides and their subsequent regioselective opening has been utilized in the formation of iminosugars such as the seven-membered ring azepanes (Assiego *et al.*, 2004), pipecolic acid derivatives (Pino-González *et al.*,2008) and piperidines (Pino-González *et al.*, 2003). In order to extend this methodology the reaction of azido ribose derivative 1 with *N*,*N*-diethyl-2-(dimethylsulfuranylidene)acetamide was investigated.

Reaction of azido ribose derivative 1 with the sulfur ylid gave the title compound, furan 3, as the major product (Fig. 1). The product was confirmed, by both X-ray crystallography and the use of D-ribose as the starting material, to have the D-*glycero*-D-*manno* stereochemistry (Fig. 2) arising from initial attack of the ylid on the *Si* face of the aldehyde, as predicted from a Felkin-Ahn model (Valpuesta Fernández *et al.*, 1990; Valpuesta *et al.*, 1993), resulting in formation of epoxide 2, followed by intramolecular opening of the epoxide to give the title compound 3.

The compound was seen to adopt weakly (O—H···N) hydrogen bonded chains of molecules running parallel to the *a*-axis. The hydrogen bond is bifurcated (Fig. 3). Only classical hydrogen bonding has been considered.

### Experimental

The title compound was recrystallized by vapour diffusion from a mixture of ethyl acetate and cyclohexane: m.p. 371–373 K; [α]_D_^23^ +16.4 (*c*, 1.0 in CHCl_3_).

### Refinement

In the absence of significant anomalous scattering, Friedel pairs were merged and the absolute configuration was assigned from the starting material.

The relatively large ratio of minimum to maximum corrections applied in the multiscan process (1:1.16) reflects changes in the illuminated volume of the crystal. Changes in illuminated volume were kept to a minimum, and were taken into account (Görbitz, 1999) by the multi-scan inter-frame scaling (*DENZO*/*SCALEPACK*, Otwinowski & Minor, 1997).

The refinement was performed excluding the data for which I was less than 3σ(I).

The H atoms were all located in a difference map, but those attached to carbon atoms were repositioned geometrically. The H atoms were initially refined with soft restraints on the bond lengths and angles to regularize their geometry (C—H in the range 0.93–0.98, O—H = 0.82 Å) and *U*_iso_(H) (in the range 1.2–1.5 times *U*_eq_ of the parent atom), after which the positions were refined with riding constraints.

### Crystal data

**Table:**

C_15_H_26_N_4_O_5_	*F*(000) = 736
*M**_r_* = 342.40	*D*_x_ = 1.232 Mg m^−^^3^
Orthorhombic, *P*2_1_2_1_2_1_	Mo *K*α radiation, λ = 0.71073 Å
Hall symbol: P 2ac 2ab	Cell parameters from 2356 reflections
*a* = 8.6440 (1) Å	θ = 5–27°
*b* = 13.4195 (2) Å	µ = 0.09 mm^−^^1^
*c* = 15.9146 (3) Å	*T* = 150 K
*V* = 1846.06 (5) Å^3^	Plate, colourless
*Z* = 4	0.60 × 0.60 × 0.40 mm

### Data collection

**Table:**

Area diffractometer	2077 reflections with *I* > 2σ(*I*)
graphite	*R*_int_ = 0.077
ω scans	θ_max_ = 27.5°, θ_min_ = 5.1°
Absorption correction: multi-scan (*DENZO*/*SCALEPACK*; Otwinowski & Minor, 1997)	*h* = −11→11
*T*_min_ = 0.82, *T*_max_ = 0.96	*k* = −17→17
23123 measured reflections	*l* = −20→20
2354 independent reflections	

### Refinement

**Table:**

Refinement on *F*^2^	Primary atom site location: structure-invariant direct methods
Least-squares matrix: full	Hydrogen site location: inferred from neighbouring sites
*R*[*F*^2^ > 2σ(*F*^2^)] = 0.049	H-atom parameters constrained
*wR*(*F*^2^) = 0.129	Method = Modified Sheldrick *w* = 1/[σ^2^(*F*^2^) + (0.1*P*)^2^ + 0.29*P*], where *P* = [max(*F*_o_^2^,0) + 2*F*_c_^2^]/3
*S* = 1.02	(Δ/σ)_max_ = 0.0003
1992 reflections	Δρ_max_ = 0.24 e Å^−^^3^
217 parameters	Δρ_min_ = −0.20 e Å^−^^3^
0 restraints	

### Fractional atomic coordinates and isotropic or equivalent isotropic displacement parameters (Å^2^)

**Table:**

	*x*	*y*	*z*	*U*_iso_*/*U*_eq_	
O1	0.35653 (19)	0.34433 (12)	0.66423 (11)	0.0350	
C2	0.3014 (3)	0.24404 (17)	0.66153 (15)	0.0332	
C3	0.4224 (3)	0.18015 (18)	0.70965 (16)	0.0371	
O4	0.3791 (2)	0.17151 (16)	0.79595 (11)	0.0446	
C5	0.4967 (3)	0.2129 (2)	0.84827 (18)	0.0489	
O6	0.5741 (3)	0.28402 (17)	0.79711 (13)	0.0567	
C7	0.5654 (3)	0.2496 (2)	0.71213 (16)	0.0414	
C8	0.5218 (3)	0.33863 (19)	0.65738 (15)	0.0359	
C9	0.5726 (3)	0.32292 (19)	0.56645 (15)	0.0369	
N10	0.5219 (3)	0.40493 (18)	0.51111 (14)	0.0432	
N11	0.3807 (3)	0.40751 (17)	0.49594 (14)	0.0432	
N12	0.2579 (3)	0.4190 (2)	0.47516 (19)	0.0629	
C13	0.4212 (5)	0.2678 (3)	0.9201 (2)	0.0714	
C14	0.6054 (4)	0.1311 (3)	0.8787 (2)	0.0650	
C15	0.4486 (3)	0.0783 (2)	0.6708 (2)	0.0472	
C16	0.1375 (3)	0.23817 (17)	0.69753 (16)	0.0343	
O17	0.0763 (2)	0.14313 (12)	0.67786 (11)	0.0401	
C18	0.0305 (3)	0.31779 (18)	0.65939 (15)	0.0334	
O19	−0.0340 (2)	0.29828 (14)	0.59206 (12)	0.0424	
N20	0.0093 (2)	0.40342 (15)	0.70067 (14)	0.0364	
C21	−0.0987 (3)	0.47788 (19)	0.66655 (18)	0.0407	
C22	−0.0208 (4)	0.5595 (3)	0.6178 (2)	0.0626	
C23	0.0809 (3)	0.4266 (2)	0.78247 (16)	0.0430	
C24	−0.0166 (4)	0.3904 (3)	0.85556 (18)	0.0562	
H21	0.2943	0.2241	0.6020	0.0397*	
H71	0.6551	0.2162	0.6930	0.0485*	
H81	0.5759	0.3970	0.6784	0.0443*	
H91	0.5284	0.2612	0.5394	0.0490*	
H92	0.6863	0.3164	0.5677	0.0495*	
H131	0.4998	0.2997	0.9504	0.1074*	
H132	0.3617	0.2267	0.9575	0.1071*	
H133	0.3543	0.3189	0.9005	0.1069*	
H141	0.7116	0.1505	0.8701	0.1022*	
H142	0.5918	0.1211	0.9387	0.1023*	
H143	0.5851	0.0695	0.8476	0.1021*	
H151	0.5319	0.0397	0.6952	0.0807*	
H152	0.3501	0.0445	0.6815	0.0799*	
H153	0.4686	0.0848	0.6104	0.0790*	
H161	0.1438	0.2430	0.7599	0.0419*	
H211	−0.1602	0.4457	0.6266	0.0494*	
H212	−0.1637	0.5025	0.7098	0.0491*	
H222	−0.1025	0.5966	0.5865	0.1080*	
H221	0.0567	0.5295	0.5787	0.1079*	
H223	0.0279	0.6004	0.6627	0.1076*	
H232	0.1785	0.3960	0.7850	0.0507*	
H231	0.0915	0.4974	0.7838	0.0496*	
H243	0.0323	0.4135	0.9063	0.0898*	
H242	−0.0188	0.3185	0.8585	0.0891*	
H241	−0.1201	0.4187	0.8522	0.0890*	
H171	0.0318	0.1201	0.6319	0.0671*	

### Atomic displacement parameters (Å^2^)

**Table:**

	*U*^11^	*U*^22^	*U*^33^	*U*^12^	*U*^13^	*U*^23^
O1	0.0338 (8)	0.0344 (8)	0.0368 (8)	−0.0019 (7)	0.0028 (7)	−0.0011 (7)
C2	0.0358 (12)	0.0336 (11)	0.0303 (10)	−0.0013 (9)	−0.0010 (9)	−0.0023 (9)
C3	0.0367 (12)	0.0413 (12)	0.0333 (11)	0.0024 (10)	0.0011 (10)	0.0029 (10)
O4	0.0404 (9)	0.0598 (11)	0.0336 (9)	0.0000 (9)	−0.0027 (8)	0.0075 (8)
C5	0.0477 (15)	0.0614 (17)	0.0376 (12)	0.0017 (13)	−0.0068 (12)	0.0052 (12)
O6	0.0642 (13)	0.0683 (13)	0.0375 (9)	−0.0153 (11)	−0.0124 (10)	0.0036 (9)
C7	0.0361 (12)	0.0505 (14)	0.0375 (12)	−0.0017 (12)	−0.0042 (10)	−0.0010 (11)
C8	0.0316 (11)	0.0403 (12)	0.0358 (11)	−0.0059 (10)	0.0019 (10)	−0.0031 (11)
C9	0.0333 (11)	0.0418 (12)	0.0357 (11)	−0.0024 (10)	0.0015 (10)	0.0000 (10)
N10	0.0422 (12)	0.0454 (12)	0.0421 (11)	−0.0054 (10)	0.0024 (10)	0.0055 (9)
N11	0.0482 (14)	0.0450 (12)	0.0365 (10)	0.0012 (10)	0.0022 (10)	0.0005 (9)
N12	0.0513 (16)	0.080 (2)	0.0577 (15)	0.0105 (15)	−0.0074 (13)	0.0077 (15)
C13	0.079 (2)	0.092 (3)	0.0433 (15)	0.010 (2)	−0.0073 (17)	−0.0083 (16)
C14	0.0592 (19)	0.077 (2)	0.0588 (18)	0.0085 (17)	−0.0179 (17)	0.0139 (17)
C15	0.0442 (14)	0.0386 (12)	0.0586 (16)	0.0043 (12)	0.0040 (13)	0.0010 (12)
C16	0.0347 (12)	0.0340 (11)	0.0342 (10)	−0.0023 (10)	−0.0002 (10)	−0.0002 (10)
O17	0.0411 (9)	0.0356 (8)	0.0437 (9)	−0.0075 (8)	−0.0057 (8)	0.0017 (7)
C18	0.0286 (10)	0.0371 (11)	0.0345 (11)	−0.0013 (9)	0.0011 (9)	0.0013 (9)
O19	0.0397 (10)	0.0462 (9)	0.0413 (9)	0.0055 (8)	−0.0070 (8)	−0.0070 (8)
N20	0.0348 (10)	0.0377 (10)	0.0366 (9)	0.0027 (8)	−0.0017 (8)	−0.0055 (8)
C21	0.0356 (12)	0.0413 (12)	0.0451 (13)	0.0055 (10)	−0.0023 (11)	−0.0043 (11)
C22	0.063 (2)	0.0606 (19)	0.0639 (19)	0.0157 (16)	0.0146 (17)	0.0167 (16)
C23	0.0496 (15)	0.0401 (12)	0.0393 (13)	0.0020 (12)	−0.0046 (12)	−0.0080 (11)
C24	0.070 (2)	0.0591 (17)	0.0393 (13)	0.0089 (16)	0.0069 (14)	−0.0047 (13)

### Geometric parameters (Å, °)

**Table:**

O1—C2	1.429 (3)	C14—H142	0.972
O1—C8	1.435 (3)	C14—H143	0.979
C2—C3	1.554 (3)	C15—H151	0.968
C2—C16	1.530 (3)	C15—H152	0.979
C2—H21	0.986	C15—H153	0.982
C3—O4	1.428 (3)	C16—O17	1.415 (3)
C3—C7	1.548 (4)	C16—C18	1.538 (3)
C3—C15	1.517 (4)	C16—H161	0.997
O4—C5	1.427 (4)	O17—H171	0.882
C5—O6	1.422 (4)	C18—O19	1.236 (3)
C5—C13	1.509 (5)	C18—N20	1.336 (3)
C5—C14	1.524 (4)	N20—C21	1.471 (3)
O6—C7	1.431 (3)	N20—C23	1.475 (3)
C7—C8	1.526 (4)	C21—C22	1.501 (4)
C7—H71	0.946	C21—H211	0.934
C8—C9	1.527 (3)	C21—H212	0.947
C8—H81	0.972	C22—H222	0.998
C9—N10	1.476 (3)	C22—H221	0.999
C9—H91	1.009	C22—H223	0.994
C9—H92	0.987	C23—C24	1.516 (4)
N10—N11	1.245 (4)	C23—H232	0.940
N11—N12	1.122 (4)	C23—H231	0.955
C13—H131	0.936	C24—H243	0.962
C13—H132	0.960	C24—H242	0.967
C13—H133	0.950	C24—H241	0.973
C14—H141	0.964		
			
C2—O1—C8	106.25 (18)	C5—C14—H142	109.8
O1—C2—C3	106.27 (19)	H141—C14—H142	107.0
O1—C2—C16	110.26 (19)	C5—C14—H143	109.7
C3—C2—C16	114.23 (19)	H141—C14—H143	109.0
O1—C2—H21	107.7	H142—C14—H143	111.0
C3—C2—H21	111.4	C3—C15—H151	115.5
C16—C2—H21	106.8	C3—C15—H152	102.5
C2—C3—O4	110.0 (2)	H151—C15—H152	109.3
C2—C3—C7	102.59 (19)	C3—C15—H153	110.2
O4—C3—C7	103.5 (2)	H151—C15—H153	108.0
C2—C3—C15	113.4 (2)	H152—C15—H153	111.4
O4—C3—C15	110.9 (2)	C2—C16—O17	108.00 (19)
C7—C3—C15	115.7 (2)	C2—C16—C18	111.91 (19)
C3—O4—C5	110.1 (2)	O17—C16—C18	108.32 (19)
O4—C5—O6	105.2 (2)	C2—C16—H161	108.6
O4—C5—C13	108.9 (3)	O17—C16—H161	107.5
O6—C5—C13	108.1 (3)	C18—C16—H161	112.4
O4—C5—C14	110.1 (3)	C16—O17—H171	131.5
O6—C5—C14	112.1 (3)	C16—C18—O19	117.8 (2)
C13—C5—C14	112.2 (3)	C16—C18—N20	119.1 (2)
C5—O6—C7	107.4 (2)	O19—C18—N20	123.1 (2)
C3—C7—O6	105.1 (2)	C18—N20—C21	119.3 (2)
C3—C7—C8	105.0 (2)	C18—N20—C23	123.9 (2)
O6—C7—C8	107.4 (2)	C21—N20—C23	116.7 (2)
C3—C7—H71	111.2	N20—C21—C22	113.7 (2)
O6—C7—H71	114.5	N20—C21—H211	107.4
C8—C7—H71	112.9	C22—C21—H211	104.0
C7—C8—O1	104.13 (19)	N20—C21—H212	110.2
C7—C8—C9	111.2 (2)	C22—C21—H212	112.8
O1—C8—C9	111.5 (2)	H211—C21—H212	108.5
C7—C8—H81	108.4	C21—C22—H222	107.7
O1—C8—H81	114.2	C21—C22—H221	109.2
C9—C8—H81	107.4	H222—C22—H221	111.3
C8—C9—N10	112.2 (2)	C21—C22—H223	102.8
C8—C9—H91	114.2	H222—C22—H223	112.6
N10—C9—H91	104.2	H221—C22—H223	112.7
C8—C9—H92	106.3	N20—C23—C24	112.1 (2)
N10—C9—H92	112.0	N20—C23—H232	108.8
H91—C9—H92	108.2	C24—C23—H232	109.1
C9—N10—N11	115.3 (2)	N20—C23—H231	105.7
N10—N11—N12	171.3 (3)	C24—C23—H231	110.8
C5—C13—H131	107.4	H232—C23—H231	110.3
C5—C13—H132	114.9	C23—C24—H243	107.2
H131—C13—H132	109.5	C23—C24—H242	111.6
C5—C13—H133	111.5	H243—C24—H242	106.8
H131—C13—H133	106.3	C23—C24—H241	110.1
H132—C13—H133	107.0	H243—C24—H241	109.0
C5—C14—H141	110.3	H242—C24—H241	112.0

### Hydrogen-bond geometry (Å, °)

**Table:**

*D*—H···*A*	*D*—H	H···*A*	*D*···*A*	*D*—H···*A*
C9—H91···O19^i^	1.01	2.30	3.140 (4)	140
C9—H92···O19^ii^	0.99	2.46	3.441 (4)	172
C23—H232···O1	0.94	2.56	3.231 (4)	129
C23—H231···O17^iii^	0.95	2.51	3.269 (4)	136
O17—H171···N10^iv^	0.88	2.30	3.112 (4)	152
O17—H171···N11^iv^	0.88	2.45	3.313 (4)	167

Symmetry codes: (i) *x*+1/2, −*y*+1/2, −*z*+1; (ii) *x*+1, *y*, *z*; (iii) −*x*, *y*+1/2, −*z*+3/2; (iv) *x*−1/2, −*y*+1/2, −*z*+1.
